# Health-related quality of life in mucopolysaccharidosis: looking beyond biomedical issues

**DOI:** 10.1186/s13023-016-0503-2

**Published:** 2016-08-26

**Authors:** Christian J. Hendriksz, Kenneth I. Berger, Christina Lampe, Susanne G. Kircher, Paul J. Orchard, Rebecca Southall, Sarah Long, Stephen Sande, Jeffrey I. Gold

**Affiliations:** 1 Adult Inherited Metabolic Disorders, Consultant Transitional Metabolic Medicine, The Mark Holland Metabolic Unit, Salford Royal NHS Foundation Trust, Ladywell NW2- 2nd Floor Room 112, Salford Manchester, M6 8HD UK; 2 Division of Pulmonary, Critical Care and Sleep Medicine, New York University School of Medicine and André Cournand Pulmonary Physiology Laboratory, Bellevue Hospital, New York, USA; 3 Centre for Rare Diseases, Clinic for children and adolescents, Helios Dr. Horst Schmidt Kliniken, Wiesbaden, Germany; 4 Institute of Medical Chemistry and Medical Genetics, Medical University of Vienna, Vienna, Austria; 5 Department of Pediatrics, Division of Blood & Marrow Transplantation, University of Minnesota, Minneapolis, MN USA; 6 GB Prohealth Ltd, Lichfield, UK; 7 School of Sociology and Social Policy, University of Bath, Bath, UK; 8 BioMarin Pharmaceutical Inc., Novato, CA USA; 9 Keck School of Medicine, Departments of Anesthesiology, Pediatrics, and Psychiatry & Behavioral Sciences, Children’s Hospital Los Angeles, Anesthesiology Critical Care Medicine, Pediatric Pain Management Clinic, University of Southern California, California, USA; 10 Paediatrics and Child Health, University of Pretoria, Steve Biko Academic Unit, Pretoria, South Africa

**Keywords:** Mucopolysaccharidoses, Quality of life, Enzyme replacement therapy, Clinical trial, Pain measurement, EQ-5D, MPS HAQ, HRQoL, ADL

## Abstract

The mucopolysaccharidoses (MPS) comprise a heterogeneous family of rare, genetic lysosomal storage disorders that result in severe morbidity and reduced life expectancy. Emerging treatments for several of these disorders have triggered the search for clinically relevant biomarkers and clinical markers associated with treatment efficacy in populations and individuals. However, biomedical measures do not tell the whole story when characterizing a complex chronic disorder such as MPS. Health-related quality of life (HRQoL) tools that utilize patient reported outcomes to address patient parameters such as symptoms (pain, fatigue, psychological health), functioning (activity and limitations), or quality of life, have been used to supplement traditional biomedical endpoints. Many of these HRQoL tools have demonstrated that quality of life is negatively impacted in patients with MPS. There is both the opportunity and need to formally standardize and validate HRQoL tools for the different MPS disorders.

**Electronic supplementary material:**

The online version of this article (doi:10.1186/s13023-016-0503-2) contains supplementary material, which is available to authorized users.

## Background

The mucopolysaccharidoses (MPS) are a group of lysosomal storage disorders associated with accumulation of glycosaminoglycans (GAGs) in tissues and organs due to enzyme deficiencies required for degradation of cellular GAGs (Additional file 1). Shared clinical features of the MPS disorders include skeletal deformities such as kyphosis, scoliosis, pectus carinatum, valgus deformities of the knees, and carpal tunnel syndrome, joint abnormalities, spinal cord compression, reduced growth, coarse facial features, vision and hearing damage, and cardiorespiratory manifestations [1, 2]. Intellectual and neurological impairment occurs in some MPS subtypes (I, II, III, and VII) due to GAG accumulation in the brain [1, 2]. MPS patients generally appear healthy at birth with clinical manifestations gradually worsening with age. There is a wide variety of clinical presentations and progression rates among the different MPS subtypes, and within the diseases themselves. Many of these disorders lead to severe morbidity and premature death [3].

Therapies including enzyme replacement therapy (ERT) and hematopoietic stem cell transplantation (HSCT) have altered the course of morbidity and mortality for some of the MPS disorders [4, 5]. Biochemical and clinical measures assessing clinical benefit for regulatory purposes have been well characterized for several of the MPS disorders and include urine GAG metabolites, 6-min walk test and pulmonary function tests. What these biomedical endpoints mean in daily life to patients and their caregivers is less well understood; and payers are increasingly asking for evidence that these treatments are having an effect that is “meaningful” to both patients and families.

The term “quality of life” is an expansive multi-dimensional concept that typically includes subjective assessments of both positive and negative aspects of life [6]. While health is an important facet of overall quality of life, it is not the only one. Other aspects, including occupation, environs, school, ethos, beliefs, and spirituality are important domains of quality of life and add to the inherent difficulty of its measurement. The concept of “health-related quality of life (HRQoL),” specifically comprises those areas of quality of life that can clearly be shown to affect health – physical, mental, emotional, and social functioning. In fact, the U.S. Centers for Disease Control has defined HRQoL as “an individual’s or group’s perceived physical and mental health over time” [7]. It should be noted that HRQoL is usually measured through self-assessment; however, if the patient is too ill or too young, a caregiver/parent assessment can serve as proxy.

In many disease states, HRQoL tools that attempt to measure patient or caregiver outcomes, are used to supplement traditional measures of morbidity, mortality and the effects of treatment. The purpose of this review is to survey the HRQoL tools that have been used to study MPS disorders, and to examine the impact of treatments on patient reported outcomes (PROs).

## Methodology

Relevant literature was obtained from clinical trial publications and PubMed searches for MeSH terms “(quality of life[MeSH Terms]) AND mucopolysaccharidoses[MeSH Terms]” (30 articles) and free text “(mucopolysaccharidosis) AND [(quality of life) or (pain) or (fatigue)]” (151 articles). Additional publications were identified from reference lists within the most relevant MPS-related papers focusing on PROs, fatigue, pain, and HRQoL. The literature search was completed in June 2015.

## How does MPS affect HRQoL?

**Fig. 1:**
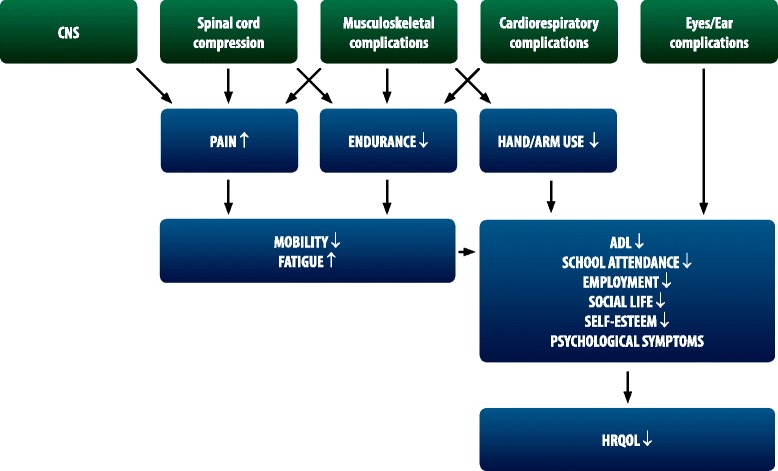
Important factors affecting HRQoL in patients with MPS. Some of the manifestations may also have a direct impact on ADL, participation in school/employment or social life (due to e.g. surgery, cognitive impairment)

The multi-organ clinical manifestations of MPS can lead to poor endurance and mobility, often associated with pain, restricted range of motion (ROM), low energy levels, and fatigue which negatively affect HRQoL and activities of daily living (ADL) (Fig. 1). MPS patients may experience increased physical and emotional dependence on family and friends, reduced participation in school, work and social life, low self-esteem, and psychological, behavioral and mental health conditions such as anxiety and depression (Fig. 1) [8]. Impaired vision and hearing and frequent surgeries may further reduce physical activity, while negatively affecting interpersonal functioning, social life, educational engagement, employment, and the ability to live independently [9–12] (Fig. 1).

**Fig. 2:**
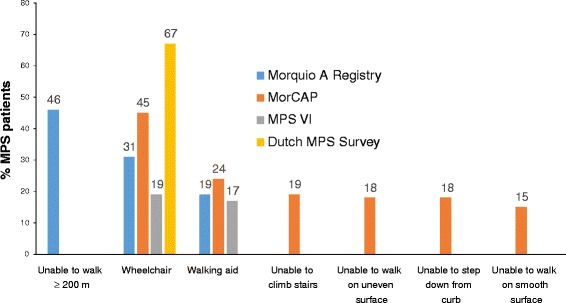
Mobility impairment in the International Morquio A registry (including 326 patients with MPS IVA) [14], the Morquio A Clinical Assessment Program (MorCAP) (including 325 patients with MPS IVA) [11], the MPS VI Survey (including 121 patients with MPS VI) [13] and the Dutch MPS Survey (including 55 patients with MPS I, II, III, IV, and VI) [15]

Impaired mobility is prevalent in MPS patients, with many individuals requiring walking aids or a wheelchair [11, 13–15] (Fig. 2). Mobility problems may be due to skeletal and joint abnormalities, spinal cord compression, pain in the lower extremities, and reduced energy levels caused by cardiorespiratory issues [2]. Joint abnormalities can result in poor shoulder ROM, wrist weakness, stiffness or changes in mobility which in turn affect simple ADL tasks such as dressing, washing and eating [16]. Pain may arise from joint defects, infections including otitis media, neurological involvement and neuropathic signals arising in the brain, increased intracranial pressure, spinal cord compression, or carpal tunnel syndrome [13, 15, 17]. Fatigue, the result of impaired cardiopulmonary function, can produce stress, anger, frustration, and potentially depression [18].

## Patient Reported Outcome (PRO) measures in MPS

PRO measures are collected by standardized questionnaires designed to measure explicit concepts such as symptoms (pain, fatigue, psychological health), functioning (activity limitations), HRQoL, or quality of life (QoL) [19]. Thousands of PRO instruments have been described including both generic and disease-specific questionnaires [20, 21]. The advantage of generic questionnaires lies in their broad applicability across different disease types, severities and medical interventions, and among diverse demographic and cultural groups, allowing comparison across studies and diseases [22]. Disease-specific questionnaires are intended for a particular patient population with questions designed to be relevant, meaningful and acceptable for that affected population, and may be used to measure the efficacy of interventions and treatments. PROs used in clinical trials with MPS patients are summarized in Table 1 and in Additional file 2, including information regarding age ranges, outcomes, and type of respondent.

**Table 1:** PROs used in patients with MPS

Name questionnaire	Acronym	Age range (yrs)	Assessment of	Completed by^a^	Reference
Symptom PROs					
Pain Visual Analog Scales^b^	VAS	≥8	Pain intensity	Patient	
Adolescent Pediatric Pain Tool	APPT	8–17	Pain location, description and intensity	Patient	[60, 61]
Brief Pain Inventory Short Form	BPI-SF	Adults	Severity of pain, impact of pain on daily function, location of pain, use of pain medications, amount of pain relief	Patient	[30, 62]
Six-face Faces Pain Scale-Revised	FPS-R	≥8	Pain intensity	Patient	[15]
Non-communicating Children’s pain Checklist-Revised	NCCPC-R	3–18^c^	Pain-associated behavior	Observer	[15, 63]
Achenbach System of Empirically Based Assessment Adult Self Report	ASEBA ASR	18–59	Social-adaptive and psychological symptoms	Patient	[30]
Achenbach System of Empirically Based Assessment Older Adult Self Report	ASEBA OASR	≥60	Social-adaptive and psychological symptoms	Patient	[30]
Yatabe-Guilford Personality test^d^	Y-G test	NA	personality and psychiatric aspects	Patient	[31]
Tree-drawing test (Baum test)	TDT	NA	personality and psychiatric aspects	Patient	[31]
General Health Questionnaire 60	GHQ-60	Adolescents and adults	Mental health	Patient	[31]
State-Trait Anxiety Inventory	STAI	Adolescents and adults	Anxiety	Patient	[31]
Functioning PROs					
Health Assessment Questionnaire	HAQ	>18	Functional capacity and independence in activities of daily living, pain, overall well-being	Patient	[13, 64]
Childhood Health Assessment Questionnaire	CHAQ	≤18		Patient	[13, 65]
Mucopolysaccharidosis Health Assessment Questionnaire	MPS HAQ	Children and adults	Self-care, mobility skills, extent of caregiver assistance in performing activities	Patient	[32]
Hunter Syndrome-Functional Outcomes for Clinical Understanding Scale	HS-FOCUS	Children (>12) and adults	Impact of MPS II on function	Patient	[27]
Modified version of the Functional Independence Measure^e^	FIM	Children and adults	Physical and cognitive disability	Observer	[31, 34]
Pediatric Evaluation of Disability inventory	PEDI	0.5–7.5	Capability and performance in self-care, mobility and social function	Patient or parent^f^	[66, 67]
Vineland Adaptive Behavior Scales	VABS	Children and adults	Adaptive behavior	Parent/caregiver	[36, 37, 39, 40]
Behavior Assessment System for Children	BASC	Children	Emotional adjustment and adaptive behavior	Patient and/or parent	[37]
Scales of Independent Behavior-Revised	SIB-R	Infancy-80+	Adaptive behavior	Patient	[68]
Health-related quality of life					
EuroQol 5D	EQ-5D	Versions for children (≥8) and adults (≥16)	Physical and mental health	Patient	[28, 69]
Short form-36	SF-36	≥16	Physical and mental health	Patient	[15, 30, 70]
Health Utilities Index	HUI	≥5	Impact of disease and therapy	Patient or parent	[27]
Pediatric Quality of Life inventory	PedsQL	Children	physical, emotional, social, and school functioning	Patient or parent	[15, 43, 71]
TNO-AZL Preschool children Quality of Life	TAPQOL	0.5–5	Physical, social, cognitive, and emotional functioning	Parent	[72]
TNO-AZL Children Quality of Life	TACQOL	6–15	Health status and children’s subjective emotional appraisal of their health	Patient or parent	[73]
Childhood Health Questionnaire	CHQ	5–18	functional capacity and independence in activities of daily life	Patient and parent	[27, 37, 39]
Impact on family/caregivers					
Pediatric Quality of Life inventory Family Impact Module	PedsQL Family Impact Module	Children	Parent’s problems in physical, emotional, social, and cognitive functioning, communication, worry, and problems specific to the family’s daily activities and family relationships	Parent	[74]
Zarit Burden Interview	ZBI	Adult patients	Burden of caring on relationship, emotional well-being, social and family life, finances, control over one’s life	Parent/caregiver	[8]

^a^In MPS studies

^b^Pain VAS scores that have been used in MPS patients are included in the HAQ, CHAQ, EQ-5D, APPT

^c^Patients who are unable to speak because of intellectual impairments or disabilities

^d^Japanese version of the Guilford test

^e^Adapted for patients with MPS

^f^Normally completed by parent or observer

*NA* not available

## Disease impact on PROs in MPS

### Impact of MPS on self-reported symptoms: pain, fatigue and psychological health

Pain has been assessed as an exploratory endpoint in several clinical trials evaluating ERT, mostly using the (Childhood) Health Assessment Questionnaire ((C)HAQ) Pain Scale or a modified version [23–26]. Baseline pain measurements from these trials recorded before patients were treated with ERT, and data from a number of other studies using (C)HAQ (Tables 2 and 3), indicate that MPS patients can experience considerable pain [23]. A score of 0.93 on a scale from 0 (no pain) to 3 (severe pain) has been reported for untreated patients with MPS I (*N* = 30) [23]. Mean Pain Scale scores reported for patients with MPS VI in the phase II study and the MPS VI Survey Study varied between 30 and 40 on a scale from 0 to 100, corresponding with mild to moderate pain, while scores were somewhat higher in older patients (>18 years) [13, 25]. A score of 28 has been reported for patients with attenuated MPS II [27].

**Table 2:** Clinical studies assessing PROs in patients with MPS, excluding ERT trials

Reference	MPS type	N	Age (yrs)	PRO instrument
[40]	MPS IH	41	NA^a^	VABS
[39]	MPS IH	47	Mean 10.5	VABS II CHQ
[34]	MPS II	27	5–41	FIM
[31]	MPS II	10	Mean 23.2	FIM Personality tests: Y-G test, Tree-drawing test Psychological tests: GHQ-60, STAI
[38]	MPS II	50	Mean 6.0	Different standardized tests for cognitive, adaptive, language, and motor functions
[35]	MPS II	29	Mean 11.5	MPS HAQ
[27]	MPS II	96 patients & caregivers	Mean 14.2	CHAQ HS-FOCUS^b^ CHQ HUI3
[36, 43]	MPS II	73 patients & parents	Mean 12.5	PedsQL Peds QL Family Impact Module VABS II
[37]	MPS II	15	10.8	VABS II CHQ BASC-2
[14]	MPS IVA	326	1–73	(C)HAQ
[11]	MPS IVA	325	Mean 14.5	MPS HAQ
[8, 28]	MPS IVA	63 patients 56 caregivers	5–17 years (*N* = 36) ≥18 years (*N* = 27)	Patients: EQ-5D, APPT (<18 years)/BPI-SF (≥18 years), fatigue question Caregivers : caregiver questionnaire, ZBI
[30]	MPS IVA	20	NA	ASEBA ASR/ OASR SF-36 BPI
[41]	MPS VIA	24	10–17 (*N* = 10) 18–54 (*N* = 14)	EQ-5D
[13]	MPS VI	121^c^	4–56	(C)HAQ
[15]	MPS I, II, III, IV, VI	55	Median 11.3	MPS-specific questionnaire NCCPC-R^d^ FPS-R^d^ Pain VAS^d^ SF-36^d^ PedsQL
[42]	MPS I, II, IVA, IVB, VI	81	≥18	EQ-5D

^a^mean age at transplant was 21.7 months; mean years of follow-up from transplant was 67.2 months

^b^HS-FOCUS completed by 53 patients aged ≥12 years

^c^Disability, Pain and Arthritis scores for 91, 90, and 81 patients ≤18 years, respectively and Disability and Pain scores for 29 and 28 patients >18 years, respectively

^d^NCCPC-R was completed by parents of patients <8 years or with intellectual disability (*N* = 35) and the FPS-R by patients 8–18 years with no intellectual disability (*N* = 11); eight patients completed the Pain VAS, 16 patients over 18 years completed the SF-36; 35 participants (patients or parents) completed the PedsQL

*NA* not available

**Table 3:** Clinical studies assessing the impact of ERT on PROs in MPS patients

Reference	MPS type	Treatment	Comparator	N	Mean age (years)^a^	Study duration	PRO instrument
[33]	I 83 % Hurler-Scheie, 13 % Scheie	iv laronidase (0.58 mg/kg/week)	Placebo	45	15.6	26 weeks	(C)HAQ
[23]	I	iv laronidase (0.58 mg/kg/week)	/	45	15.7	3.5 year (extension of [66]	(C)HAQ
[52]	I	iv laronidase (0.58 mg/kg/week)	/	5	12.0	6 year	Modified MPS HAQ
[53]	I Scheie, Hurler- Scheie	iv laronidase (0.58 mg/kg/week)	/	7	16.3	52–208 weeks	MPS HAQ
[55]	II	iv idursulfase (0.5 mg/kg/week)	/	94	14.5	2 years extension	(C)HAQ
[32, 45]	IVA	iv elosulfase alfa (2.0 mg/kg every other week or weekly)	Placebo	176	15.3 and 13.1	24 weeks	MPS HAQ
[29]	IVA	iv elosulfase alfa (2.0 or 4.0 mg/kg/week)	/	25	13.7	27 weeks	APPT
[24]	VI	iv galsulfase (1.0 or 2.0 mg/kg/week)	/	5	11.0	48 weeks	(C)HAQ
[25]	VI	iv galsulfase (1.0 mg/kg/week)	/	10	12.7	48 weeks	(C)HAQ
[26]	VI	iv galsulfase (1.0 mg/kg/week)	Placebo	39	13.7	24 weeks	Joint pain and stiffness, physical energy level
[56]	VI	iv galsulfase (1.0 mg/kg/week)	/	9	NA	2 years	(C)HAQ
[5]	VI	iv galsulfase (1.0 mg/kg/week)	/	55	12.0	6.8 ± 2.2 years	(C)HAQ
[44]	VI	iv galsulfase (1.0 mg/kg/week)	/	8	6.8	1.0–4.5 years	TAPQOL/TACQOL^b^

^a^Mean age at baseline from all patients or from ERT group

^b^The TAPQOL was completed by the parents of four patients <6 years, TACQOL was completed by seven parents of patients ≥6 years

*NA* not available

Several studies have evaluated pain in MPS in more detail using other questionnaires [15, 28, 29]. In the Dutch National MPS Survey of 55 patients with different types of MPS, joint pain was evaluated with the Non-communicating Children’s Pain Checklist-Revised (NCCPC-R), the Six-face Faces Pain Scale-Revised (FPS-R) and an MPS-specific questionnaire. Overall, 69 % of patients reported pain, mainly hip and back pain (27.8 and 25.9 %, respectively), with a pain score above the critical cut-off value for significant pain in 40 % of cases [15]. Somewhat surprisingly, pain was most frequently reported for patients with cognitive impairment, particularly for MPS III, while patients with MPS IV (which is not associated with cognitive impairment) appeared to experience the most severe pain [15]. The unexpected high prevalence of pain in cognitively impaired patients suggests that pain may be underestimated in this group, but may also reflect difficulties with parents distinguishing between MPS- and pain-related behavior (as assessed in the NCCPC-R) in these patients.

The finding from the Dutch survey that pain was most severe in MPS IV patients is not unexpected given the severe skeletal and joint abnormalities in these individuals. Consistent with this finding, a phase II MPS IVA study reported a pain intensity score of 4.6 on the Adolescent Pediatric Pain Tool (APPT) at baseline, indicating medium pain [29]. In addition, an international MPS IVA PRO survey reported joint pain in 74 % of adults (*N* = 27) and 64 % of children (*N* = 36), as documented using the Brief Pain Inventory Short Form (BPI-SF) and APPT [28]. In both studies, pain was described most often in the lower extremities [28, 29]. The PRO survey also demonstrated an association between pain and mobility as measured by wheelchair use. Adult patients who sometimes used a wheelchair tended to report more severe and widespread pain than those always using a wheelchair, while pain interference with daily activities was highest in the latter group [28]. This suggests adult MPS IVA patients may tolerate considerable pain if mobility and wheelchair independence are retained.

Our literature search revealed only a single study assessing fatigue. The aforementioned MPS IVA PRO study, assessed fatigue/low stamina by querying patients on the number of evenings per week that they reported feeling extremely tired. Using this definition, 63 % of adults and 69 % of children reported feeling fatigued, a high prevalence warranting further investigation. Possible contributions from pulmonary or cardiac causes would be difficult to distinguish in many patients.

To date, two studies have evaluated the psychological health of patients with MPS [30, 31]. A study in ten MPS II patients showed that many had difficulties establishing relationships and that patients and their parents had increased levels of anxiety [31]. A correlation was found between psychological status and ADL, suggesting that reduced ADL negatively affects psychological status [31]. Another study of 20 MPS IVA patients showed psychological symptoms (at least one or more ASEBA [Achenbach System of Empirically Based Assessment, which assesses social-adaptive function deficits and psychological symptoms] problem Scales within the symptomatic range) in 11 individuals [30]. Interestingly, these patients had higher pain severity scores and pain interference scores on the BPI, suggesting that pain and psychological issues, including depression, may be interdependent.

### Impact of MPS on patient functionality

ADL, as assessed by the MPS Health Assessment Questionnaire (MPS HAQ), have been measured as an exploratory endpoint in some clinical trials [32, 33], as well as in a number of studies in patients with MPS II, IV and VI [11, 14, 34, 35] (Table 2). Overall difficulties with mobility and self-care, which tend to increase with age, have been reported. In patients with MPS II, cognitive decline negatively affects ADL.

A study of 96 patients with attenuated MPS II (age 5.0–30.9 years) reported impairments in walking/standing and reach/grip domains of the Hunter Syndrome-Functional Outcomes for Clinical Understanding Scale (HS-FOCUS) and impairments in hygiene, reach and dressing, and grooming domains of the CHAQ [27]. HS-FOCUS function scores were lower in patients with better endurance in the 6MWT (*r* = −0.6) and better joint mobility (*r* = −0.3). Another smaller study (*N* = 29; age 2–29 years) suggested that difficulties with ADL (as assessed using the MPS HAQ) in MPS II patients mainly depend on the cognitive status and age of these patients [35]. Younger patients with normal mental development were generally independent with regard to self-care, mobility and walking, but assistance with daily activities increased with age [35]. Cognitively impaired MPS II patients required moderate or complete caregiver assistance in self-care within all categories [35]. Two studies used the Functional Independence Measure (FIM) to assess ADL in patients with MPS II [31, 34]. In patients with severe MPS II, cognitive scores decreased rapidly, reaching a minimum score at about 7 years of age, in contrast to motor scores, which decreased more slowly. In slowly progressing MPS II patients, total FIM scores increased with age, similar to increases in FIM scores seen in healthy children [34]. In patients with MPS II, daily living skills have also been assessed as part of adaptive behavior scales [36, 37]. In both mild and severe forms of MPS II (*N* = 73), the Vineland-II Adaptive Behavior Scales (VABS II) showed significantly reduced functioning in communication, daily living skills, socialization, and motor skills as compared to normative data [36], but scores were significantly lower (more severe) in severe than in mild MPS II. A study including 15 patients with slowly progressing MPS II showed adaptive skills within the average range on the VABS II, as well as the Behavior Assessment System for Children (BASC)-2 Parent Rating Scale [37]. Daily living skills domain scores of the VABS II decreased significantly with age across patients. Children aged ≥12 years showed an increasing sense of inadequacy and anxiety as well as decreasing self-esteem over time in the BASC-2. In a retrospective review of longitudinal data from 50 patients with MPS II, two groups of patients could be distinguished based on adaptive behavior data (obtained using the Scales of Independent Behavior, Revised [SIB-R] and the Pediatric Evaluation of Disability Inventory [PEDI]): one group reaching a plateau at around 48–60 months and then declining and one group maintaining relatively normal adaptive abilities over time [38]. In patients with MPS IH, the VABS has been used to evaluate the impact of HSCT on adaptive skills [39, 40]. These studies are discussed below under “Effects of therapy on HRQoL in MPS”.

**Fig. 3:**
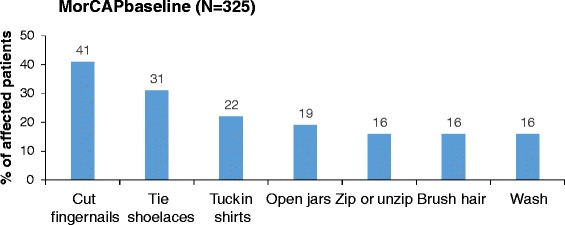
Impact of MPS on self-care ADL as measured by the MPS HAQ in the MorCAP study including 325 patients with MPS IVA (mean age 14.5 years) [11]

MPS IVA and MPS VI have also been shown to significantly interfere with patients’ ADL [14]. In the International Morquio A registry of 326 MPS IVA patients, only 40–60 % of patients were able to perform ADL independently [14]. In the MorCAP study with 325 MPS IVA patients, 20–40 % reported self-care ADL tasks (including the ability to wash or brush hair, tie shoelaces and cut fingernails) were affected by their disease (Fig. 3) [11]. In the Survey Study of 121 MPS VI patients, the (C)HAQ disability index indicated a mild level of disability in patients aged >18 years (mean 1.0) and moderate disability in those aged ≤18 years (mean 2.0) [13].

### Impact of MPS on health-related quality of life (HRQoL)

Several studies report significant disease impact on HRQoL in patients with MPS disorders (Table 2). Overall, the greatest deviations from a healthy population were seen in domains of pain/discomfort and mobility. Problems with self-care or usual activities were also critical factors affecting HRQoL. In addition, wheelchair use, unemployment, poor endurance, and poor pulmonary function were also associated with worse HRQoL [28, 41]. Despite the deviation in pain domains, no differences in HRQoL could be found between patients with or without pain [15, 28]. Although pain has been identified as a significant issue for patients with MPS, other symptoms such as mobility appear to have greater impact on HRQoL.

Two studies used the generic Short Form-36 (SF-36) to assess HRQoL in patients with MPS (Table 2). In 16 adults from the Dutch national MPS survey, including patients with MPS I, II, III, IV and VI, deviations from average were predominantly seen in the physical component score (29–30 vs. 50 in a reference population) [15]. The largest deviation was observed in the bodily pain domain (37–41 vs. 81), possibly due to bone pain reported in 68 % of patients. The Pediatric Quality of Life (PedsQL) was used in this study to assess HRQoL in patients <18 years, showing the largest deviations compared with healthy individuals in the PedsQL physical score (53–57 vs. 79–85 in a reference population). A study including 20 patients with MPS IV recently showed scores below the US mean in physical, but not mental, health on the SF-36 [30]. Although several patients had psychological symptoms on the ASEBA Adult Self-Report (ASR), these did not seem to affect HRQoL outcomes.

**Fig. 4:**
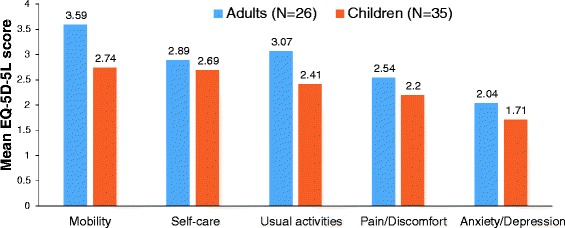
Mean score for the five EQ-5D domains in 61 MPS IVA patients (adults and children) [28]

Two studies used the generic Euroqol-5 dimensions (EQ-5D) questionnaire, assessing mobility, self-care, usual activities, pain/discomfort, and anxiety/depression in patients with MPS. Lavery et al. examined 81 adult patients from the UK and USA with various types of MPS and a mean utility value of 64.1 (with 100 indicating best health) [42]. HRQoL in these patients was mainly affected by mobility impairment and pain/discomfort, and to a lesser degree by problems with self-care or performing usual activities [42]. In the international PRO survey using the EQ-5D, Hendriksz et al. [28] reported impairment in mobility, self-care, usual activities, pain/discomfort, and anxiety/depression for both adults (*N* = 27) and children (*N* = 36) with MPS IVA (Fig. 4). Results from this study indicated that HRQoL diminished with increasing wheelchair use. Adult patients who used a wheelchair sometimes (when needed) had a mean utility value of 0.582, a score comparable to that for patients with chronic ischemic heart disease or non-insulin dependent diabetes mellitus (Table 4). The utility value of adults using a wheelchair all the time (0.057) was only slightly better than that of bed ridden or completely immobile multiple sclerosis patients (Table 4). EQ-5D utility values were also considerably lower in unemployed (0.275) than in employed (0.640) MPS patients [28]. A study in a subset of German MPS IVA patients (*N* = 24) from the international PRO survey showed strong correlations of EQ-5D utility values with endurance in the 6-min walk test and 3-min stair climb test (*R* = 0.884 and *R* = 0.852, respectively) and with pulmonary function (forced vital capacity: *R* = 0.815; maximum voluntary ventilation: *R* = 0.825), suggesting that these measures might be used as surrogate measures for HRQoL in patients with MPS IVA [41].

**Table 4:** Comparison of HRQoL according to the EQ-5D utility value in MPS IVA (Morquio A) patients with different levels of mobility impairment and other serious chronic diseases

Disease	HRQoL mean utility^a^
MPS IVA: adults, no wheelchair [28]	0.846
MPS IVA: adults, sometimes wheelchair [28]	0.582
MPS IVA: adults, always wheelchair [28]	0.057
Multiple sclerosis, walking-aid [75]	0.460
Multiple sclerosis, bedridden [75]	−0.195
Moderate to severe rheumatoid arthritis [76]	0.489
Chronic ischaemic heart disease [77]	0.640
Non-insulin dependent diabetes mellitus [77]	0.670

^a^EQ-5D utility scores were calculated using a time tradeoff method [78]. This generates scores ranging from −0.59 to 1, where 1 means full health and zero stands for death. Negative scores could be emotively interpreted as a health state “worse than death”. Tradeoff tariffs used differed depending on the ethnic background of patients

Several MPS studies have used generic instruments to measure HRQoL in children. The Dutch national MPS survey of patients with MPS I, II, III, IV and VI [15] used the PedsQL to assess HRQoL in patients <18 years, with the largest deviations compared to healthy individuals seen in the PedsQL physical score (53–57 vs. 79–85 in a reference population). The PedsQL was also used in a study of 73 patients with MPS II and their parents, showing reduced scores in all domains (physical, emotional, social, and school functioning) versus healthy individuals and patients with several other chronic illnesses (cancer, maple syrup urine disease, galactosemia) [43]. In slowly progressing MPS II patients (*N* = 96), Raluy-Callado et al. [27] demonstrated significant distress and dysfunction in global health, physical functioning and role/social-limitations-physical and bodily pain, as measured by the generic Childhood Health Questionnaire (CHQ) (*N* = 96). Low scores were reported in the self-esteem and family cohesion domains, suggesting that MPS II has a severe psychological impact on patients and their parental caregivers. It is unclear how these domains were affected by cognitive function. A clear correlation between joint ROM and better physical functioning scores of the CHQ (*r* = 0.5) was observed. A smaller study in 15 slowly progressing MPS II patients showed a CHQ psychosocial summary score within the normal range. The physical summary score was 1.5 standard deviations below the normative average for the whole group, >2 standard deviations below average in children ≥12 years (*N* = 10), and tended to worsen with age [37].

Finally, Brands et al. [44] used the TNO-AZL Child Quality of Life (TACQOL) and TNO-AZL Preschool Children’s Quality of Life (TAPQOL) questionnaires to evaluate the impact of ERT on HRQoL in children with MPS VI aged 6–15 years (*N* = 7) and aged 6 months to 6 years (*N* = 4), respectively (Table 3). Baseline data reflected the greatest deviations from healthy peers in lung problems, social functioning, motor functioning and positive mood domains of the TAPQOL and in body and motor domains of the TACQOL [44].

### Impact of MPS on caregivers

**Fig. 5:**
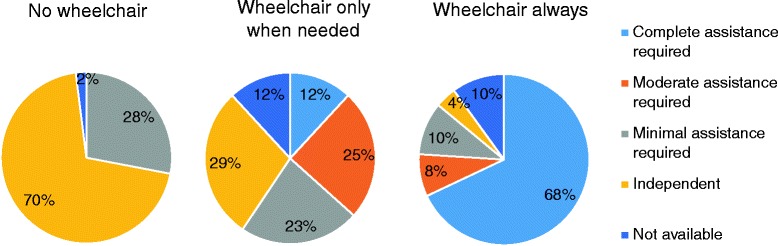
Level of assistance required from caregivers for performing daily activities as measured by the MPS HAQ in adult MPS IVA patients, according to wheelchair use [8]

Only a few studies, to date, have addressed the impact on caregivers looking after individuals with MPS. In a study including 73 caregivers of patients with MPS II (both mild and severe forms), the PedsQL Family Impact Module showed that the impact of the disease on the family is similar to that for other pediatric outpatients with chronic illnesses [36]. Domain scores for family HRQoL, family functioning summary, total scale score, physical functioning, social function, daily activities, and family relationships negatively correlated with the severity of illness. In the international PRO survey in MPS IVA patients [8], outcomes of the Zarit Burden Interview (ZBI), the MPS HAQ, and a caregiver questionnaire revealed that MPS IVA poses a large burden on caregivers, affecting their physical and emotional health, family life, social life and financial situation. Caregiver burden increased with disease progression and mobility problems. Wheelchair use by MPS IVA patients had a profound negative impact on caregiver’s support (Fig. 5) [8]. Because wheelchair-bound patients require much more caregiver support than those using a wheelchair occasionally, the investigators concluded that even small improvements in patient mobility might substantially reduce the level of caregiver support and the burden of caregiving.

## Effects of therapy on HRQoL in MPS

### Therapies for MPS

Two treatment options target the pathophysiology of MPS: HSCT and ERT. HSCT is primarily used in MPS conditions with a neurologic component since difficulties exist in delivery of intravenous enzyme products across the blood-brain barrier [9]. ERT is currently available to treat MPS I, II, IVA and VI. Several randomized, placebo-controlled phase II/III clinical studies have demonstrated favorable effects of ERT on urinary GAG levels, endurance, respiratory function, joint ROM, hepatomegaly, growth/height, and cardiac function [25, 45–49].

### HSCT

While HSCT has been considered the standard of care for the severe form of MPS I (Hurler syndrome) for decades [50], effects on patient reported HRQoL are not well studied. A few studies in patients with MPS IH and MPS II have been published.

A study of 41 MPS IH children transplanted at a mean age of 21.7 months and followed for 2–21 years (mean follow-up 67.2 months) showed declining adaptive behavior scores on the VABS over time, indicating development of skills at a lower than average rate compared with unaffected peers [40]. VABS scores were significantly better in transplanted patients after the age of 2 years when compared to a cross-sectional non-transplanted MPS IH group. Cognitive ability, not age, at transplant correlated significantly with the ultimate adaptive level. Another study of 47 MPS IH patients transplanted between 6 and 44 months and evaluated 1–24 years post-HSCT showed no significant impact of the type of transplant, number of transplants, age at transplant, time since transplant, or total body irradiation treatment on adaptive functioning on the VABS. [39]. However, individuals undergoing HSCT at an older age reported poorer physical QoL on the CHQ. In addition, patients receiving unrelated bone marrow HSCT exhibited poorer psychosocial QoL compared with those receiving bone marrow HSCT from a relative.

Finally, a retrospective study in 13 HSCT-treated, Japanese MPS II patients showed stable or improved ADL (school status, movement and daily activities, conversation, and toileting) versus baseline in most patients after a mean follow-up of 9.6 years [51].

### ERT

Several clinical trials evaluating ERT in patients with MPS I, II, IVA and VI have included PRO measures as exploratory efficacy endpoints using the (C)HAQ or MPS HAQ (Table 3). It should be noted that these studies were not powered to assess the true effect of ERT on PRO measures and results should be interpreted with caution.

#### MPS I

Studies in patients with MPS I describe improvements in ADL, pain and HRQoL after long-term ERT [23, 52, 53]. The 3.5-year extension of the laronidase phase III study (*N* = 45) showed a stable or improved (C)HAQ Disability Index in 77 % of patients (57 % improved) [23]. The mean decrease of 0.31 was considered clinically meaningful based on data from patients with rheumatoid arthritis [54]. In the 30 patients in this study with available pain data, the Pain Index decreased from 0.93 at baseline to 0.56 after long-term treatment. A smaller study of 7 patients with attenuated MPS I (Scheie or Hurler-Scheie) showed significant improvements in ADL (eating/drinking, dressing, tooth brushing, toileting, and walking) as documented by the MPS HAQ after 52–208 weeks of ERT [53]. The impact of ERT on HRQoL in patients with MPS I has been assessed in a 6-year re-evaluation of patients enrolled in the original laronidase phase I/II trial using an MPS-specific QoL questionnaire containing 100 questions. The largest effects of treatment were reported for energy, endurance, independence (personal hygiene, dressing, transfers), sleep quality, participation in daily activities, and self-esteem [52].

#### MPS II

One long-term open-label study has investigated the effect of ERT on PRO measures in patients with MPS II. This study demonstrated statistically significant improvements from baseline in the CHAQ Disability Index in 48 patients aged ≥12 years as soon as 20 months after ERT initiation [55]. Disability was also evaluated by a parent-assessed Disability Index completed by 81 parents, showing significant improvements 8 months after onset of ERT.

#### MPS IVA

Studies in patients with MPS IVA have shown improvements in ADL and pain within a relatively short time after ERT initiation [29, 32, 45]. The MPS HAQ was used to evaluate the effect of ERT on ADL in 176 patients with MPS IVA in a phase III study. After 24 weeks, small to modest improvements in caregiver assistance and mobility domains were observed for ERT compared to placebo, though with wide confidence intervals [32, 45]. Interim results of an ongoing randomized, double-blind study in 25 MPS IVA patients with relatively good endurance showed clear (numerical) improvements in pain, as assessed using the APPT, after 24 weeks of ERT [29].

#### MPS VI

Studies in patients with MPS VI have shown improvements or no change in ADL, pain and HRQoL in patients receiving ERT [5, 24–26, 44, 56]. The (C)HAQ, or a modified version, was used in all clinical trials evaluating the efficacy of ERT in MPS VI, showing improvements in pain and arthritis or joint stiffness and improvements in ADL (e.g. picking up coins, tying shoelaces, pulling shirt overhead) as measured by investigator observation in the phase I/II and phase II studies [24, 25]. In a small Taiwanese open-label study, nine patients showed an improvement in the (C)HAQ Disability Index with ERT [56]. However, these results could not be confirmed in the phase III study, which reported no changes in any of the tertiary efficacy measures [26]. Similarly, a 10-year Resurvey of the MPS VI Survey Study reported no change in the HAQ disability, pain and arthritis scores from baseline in patients receiving ERT for a mean period of 6.8 years despite the fact that most of the participants had rapidly progressing phenotypes [5]. A Dutch study of 11 patients with MPS VI showed improvements with ERT in lung problems, sleeping, liveliness, positive mood, social functioning, and communication domains of the TAPQOL in younger patients and in body and motor domains of the TACQOL in older patients [44].

## Discussion and conclusions

In recent years, more patient-centric studies have attempted to measure the burden of illness as it relates to individuals with MPS disorders. The varied clinical manifestations of MPS disorders, from skeletal, pulmonary and cardiac impairment to psychological, fatigue and pain management issues make this group of diseases unique and challenging from both the clinicians’ and patients’ perspective. The use of formal PRO tools to measure ADL and HRQoL has given researchers much insight into what patients living with a progressive, debilitating disease like MPS go through on a daily basis. While recent studies have specifically focused on PROs as clinically meaningful measures for functioning and life, PROs were previously mainly used as exploratory endpoints in clinical trials. Our review of the current literature demonstrates that HRQoL is strongly negatively affected in both MPS patients and their caregivers, with mobility, pain and psychological issues being significant problem areas. Two approved treatments for MPS are currently available, HSCT and ERT. The effects of HSCT on patient reported HRQoL has not been adequately addressed to date and future research is warranted. Multiple studies in patients with MPS I, IVA and VI receiving ERT report improvements in ADL, pain and HRQoL. Clinicians, industry, regulatory agencies and payers increasingly recognize the importance of PROs in the evaluation of new therapies. PROs should continue to be utilized in future ERT studies as primary or secondary endpoints to capture relevant data on HRQoL and there is both the opportunity and the need to validate and expand the routine use of customized MPS-specific HRQoL tools.

While PRO measures often provide important information about the burden of illness in patients with MPS, they may be limited in their use, particularly in children or adolescents. HRQoL is a multi-dimensional concept that includes subjective evaluations, which can be challenging to measure due to heterogeneity in number and content of domain items included in questionnaires, discrepancies between patient and parent ratings, and lack of information regarding test–retest reliability, structural validity, or sensitivity to change [57, 58]. It is also important to keep in mind that while functional status may be related to HRQoL, functionality may not always be indicative of a patient’s subjective perception of his or her life [57, 59]. For example, children who have never experienced a healthy state and who have adapted to this condition may have a good HRQoL despite their functional limitations [57]. In addition, the impact of physical, social, and cognitive factors on HRQoL can change with age [59]. Social roles and independence may be more important for HRQoL in adolescents than for children [59]. Therefore, the terms HRQOL, health status, and functioning should not be used interchangeably, and different types of items and response formats should be used for different ages or developmental levels. Although several generic PRO tools used in MPS studies are validated and allow comparisons across diseases, disease-specific PRO measures may be more suitable as endpoints in clinical trials as they are more likely to detect clinically meaningful changes [58, 59].

The results of PRO assessments should always be seen in the socio-cultural and economic context in which they exist and should take into account the patient’s personality, cognitive ability, and community support network. In addition, ADL measures need to become broader in scope to capture technical advances of the modern world that increasingly impact, both positively and negatively, the lives of patients. Finally, it is important to keep in mind that a patient’s medical status is only a part of his or her personality. It is hoped that QoL assessments do not provoke alienation from a patient’s own personality and curtail his or her ability to flourish. To address these challenges, health professionals and patients should develop partnerships to exchange academic and life experiences. As the MPS disorders are rare diseases and patients are spread over many centers, it will be important to develop a unified approach for monitoring PROs in these patients.

In closing, the impact of MPS on subjective symptoms, functionality, and HRQoL is a critical area of investigation in the field of lysosomal storage disorders. Development of valid and reliable assessment tools and implementation of routine evaluations could lead to early identification of areas of difficulty and subsequent intervention, minimizing the negative impact of MPS-related problems. Managing the negative effects of MPS through early identification and treatment will prove vital for both patients and caregivers. Ultimately, focusing on the primary medical disease alone is not enough when treating patients with a chronic illness. Considering the entire person’s biology, psychology, and social circumstances will ultimately lead to improved patient outcomes and a better understanding of the unique challenges they face.

## Additional files

Additional file 1: Table S1. Classification of MPS. (DOCX 13 kb) Additional file 2: Patient-reported outcome (PRO) measures used in mucopolysaccharidosis (MPS) studies. (DOCX 66 kb)

## Abbreviations

3MSC: 3-minute stair climb
6MWT: 6-minute walk test
ADL: Activities of daily living
APPT: Adolescent pediatric pain tool
ASEBA: Achenbach system of empirically based assessment
ASR: Adult self-report
BASC: Behavior assessment system for children
BPI-SF: Brief pain inventory short form
CHAQ: Childhood health assessment questionnaire
CHQ: Childhood health questionnaire
EQ-5D: Euroqol-5 dimensions
ERT: Enzyme replacement therapy
FIM: Functional independence measure
FPS-R: Six-face faces pain scale-revised
FVC: Forced vital capacity
GAG: Glycosaminoglycan
GHQ-60: General health questionnaire 60
HRQoL: Health-related quality of life
HSCT: Hematopoietic stem cell transplantation
HS-FOCUS: Hunter syndrome-functional outcomes for clinical understanding scale
HUI: Health utilities index
MCS: Mental component score
MorCAP: Morquio A clinical assessment program
MPS: Mucopolysaccharidoses
MPS HAQ: Mucopolysaccharidoses health assessment questionnaire
MVV: Maximum voluntary ventilation
NCCPC-R: Non-communicating children’s pain checklist-revised
OASR: Older adult self-report
PCS: Physical component score
PEDI: Pediatric evaluation of disability inventory
PedsQL: Pediatric quality of life
PRO: Patient-reported outcome
QoL: Quality of life
rhASB: Recombinant human arylsulfatase B
ROM: Range of motion
SF-36: Short form-36 (items)
SIB-R: Scales of independent behavior, revised
STAI: State-trait anxiety inventory
TACQOL: TNO-AZL child quality of life
TAPQOL: TNO-AZL preschool children’s quality of life
VABS: Vineland adaptive behavior scales
VAS: Visual analogue scale
Y-G test: Yatabe-Guilford personality test
ZBI: Zarit Burden interview
