# Supplementary material for: Health-related quality of life in mucopolysaccharidosis: looking beyond biomedical issues
Source: Orphanet J Rare Dis. 2016 Aug 26;11(1):119. doi: 10.1186/s13023-016-0503-2 (PMC5000418; doi:10.1186/s13023-016-0503-2)
Supplement: Additional file 1: Table S1. — Classification of MPS. (DOCX 13 kb) [file 13023_2016_503_MOESM1_ESM.docx]

**Additional Table 1:** Classification of MPS

| **MPS Type/ ORPHA number** | **Sub-categories** | **Deficient enzyme** | **Affected GAG** | **Major clinical manifestations** |
| --- | --- | --- | --- | --- |
| I/ 579 | Hurler | α-L-iduronidase | Heparan sulfate,  Dermatan sulfate | Corneal clouding, dysostosis multiplex, organomegaly, heart disease, mental retardation |
|  | Scheie |  |  |  |
|  | Hurler-Scheie |  |  |  |
| II/ 580 | Hunter | Iduronate-2-sulfatase | Heparan sulfate,  Dermatan sulfate | Mental retardation, organomegaly, short stature |
| IIIA/ 79269 | Sanfilippo A | Heparan N-sulfatase | Heparan sulfate | Mental retardation, hyperactivity |
| IIIB/ 79270 | Sanfilippo B | α-N-acetyl-glucosaminidase |  |  |
| IIIC/ 79271 | Sanfilippo C | α-glucosaminide acetyltransferase |  |  |
| IIID/ 79272 | Sanfilippo D | N-acetylglucosamine 6-sulfatase |  |  |
| IVA/ 309297 | Morquio A | N-acetylgalactose 6-sulfatase | Keratan sulfate,  Chondroitin sulfate | Skeletal abnormalities, corneal clouding, short stature, cardiorespiratory |
| IVB/ 309310 | Morquio B | β-galactosidase | Keratan sulfate |  |
| VI/ 583 | Maroteaux-Lamy | Arylsulfatase B | Dermatan sulfate, Chondroitin sulfate | Dysostosis multiplex, corneal clouding, short stature |
| VII/ 584 | Sly | β-glucuronidase | Dermatan sulfate, Heparan sulfate, Chondroitin sulfate | Hepatomegaly, dysostosis multiplex, mental retardation |
| IX/ 67041 |  | Hyaluronidase | Hyaluronan | Periarticular soft tissue masses, short stature |
