# Supplementary material for: Health-related quality of life in mucopolysaccharidosis: looking beyond biomedical issues
Source: Orphanet J Rare Dis. 2016 Aug 26;11(1):119. doi: 10.1186/s13023-016-0503-2 (PMC5000418; doi:10.1186/s13023-016-0503-2)
Supplement: Additional file 2: — Patient-reported outcome (PRO) measures used in mucopolysaccharidosis (MPS) studies. (DOCX 66 kb) [file 13023_2016_503_MOESM2_ESM.docx]

**Additional file 2:** Patient-reported outcome (PRO) measures used in mucopolysaccharidosis (MPS) studies

**Symptom PROs**

Symptom PROs assess deviations from an individual’s normal physiological condition, describing symptoms such as pain, fatigue or psychological status. Pain scales used in MPS trials include the Brief Pain Inventory Short Form (BPI-SF) [1, 2], the six-face Faces Pain Scale-revised (FPS-R) [3], and the Non-communicating Children’s Pain Checklist-Revised (NCCPC-R) [3]. These are all validated instruments used across various diseases in which pain plays a significant role [4]. The BPI-SF is used with adult patients to rate the severity and location of pain, and the impact of pain on daily functioning. It also documents use of pain medications and degree of pain relief [1].The FPS-R uses six faces to describe pain, from no pain to very much pain (score 0-100), in patients aged 8 years or older [3]. The NCCPC-R is designed for children from 3 to 18 years who are unable to speak because of intellectual impairments or disabilities and consists of a list of 30 items divided into seven subdomains (vocal, eating and sleeping, social, facial, activity, body and limb, and physiological signs) [5]. This questionnaire is completed by an observer, usually a parent, based on pain-associated behaviour. However, when used in MPS patients with behavioural problems (such as MPS III patients), it may be difficult to distinguish between MPS-specific and pain behaviour [3]. Pain scales used in MPS trials also include Visual Analog Scales (VAS) such as that of the Adolescent Pediatric Pain Tool (APPT), which also assesses pain location(s) and pain description, and HRQoL questionnaires such as the Health Assessment Questionnaire (HAQ) for adult patients, the Childhood Health Assessment Questionnaire (CHAQ) for children with arthritis, and the Quality of Life EuroQoL (EQ)-5D questionnaire [6-9]. VAS can be used easily and quickly to obtain a quantitative measure of pain, but may be less suitable for use in young children (<8 years) or patients with cognitive impairment who may have difficulty understanding its concept [10]. Information on pain and fatigue has also been obtained in several MPS clinical trials by asking simple questions about the use of pain medications or the number of evenings patients were feeling extremely tired [4, 6].

Social-adaptive and psychological symptoms have been evaluated in one study in MPS IVA patients using the Achenbach System of Empirically Based Assessment (ASEBA) Adult Self-Report (ASR) and Older Adult Self-Report (OASR) [2]. Mental health has been assessed in MPS II patients and their families using the Yatabe-Guilford Personality test (Y-G test), the Tree-Drawing Test, the General Health Questionnaire 60 (GHQ-60) and the State-Trait Anxiety Inventory (STAI) [11].

**Functioning PROs**

Functioning PROs include any physical, social or psychological restriction or lack of ability to perform an activity in a way considered normal. Examples include assessments of ADL such as dressing, walking or personal care.

The HAQ and the CHAQ, originally developed for patients (or parents of patients) with rheumatoid arthritis [12, 13], have been used in MPS patients to evaluate functional capacity and independence in ADL across eight domains: dressing and grooming, getting up, eating, walking, reach, grip, hygiene, and activities. The Disability Index represents an overall summary score of the (C)HAQ[6]. The (C)HAQ also includes two VAS scores for pain evaluation and overall well-being, which can also be used independently. The HAQ was later adapted to capture self-care, mobility skills and the extent of caregiver assistance required in patients with MPS I (MPS HAQ) [14, 15]. The MPS HAQ is a survey that assesses self‑care (eating/drinking, dressing, bathing, grooming, tooth brushing, and toileting), mobility skills (dexterity, mobility, walking, stair climbing, and gross motor skills) and the extent of required caregiver assistance in the performance of these activities. Scores of both the (C)HAQ (0-3) and MPS HAQ (1-10 or 1-4 depending on the domain)increase with increasing impairment in functionality. The Disability Index is calculated by the sum of all items in the self-care domain divided by the number of items, with a higher score indicating more impaired functionality. Although the (C)HAQ and MPS HAQ are currently the most frequently used tools to measure the impact of MPS on ADL, they are not validated for use in these patients.

Recently, the Hunter Syndrome-Functional Outcomes for Clinical Understanding Scale (HS-FOCUS) was developed to capture the impact of MPS II on function [16]. The final patient and parent versions of this questionnaire contain six domains measuring functional status, i.e. walking/standing, reach/grip, sleeping, schooling/work, activities, and breathing. Each item is scored from 0 (ability to complete the activity without any difficulty) to 4 (unable to do so). Finally, a modified version of the Functional Independence Measure (FIM) has been used in two MPS II studies [11, 17]. The FIM must be completed by an observer and measures physical (13 items) and cognitive (5 items) disability by scoring the level of assistance required for an individual to perform ADL [18]. Similar to the (C)HAQ and MPS HAQ, the HS-FOCUS and the FIM have not been validated for use in MPS patients.

Daily living skills have also been evaluated in MPS patients as part of measures of adaptive function. The Vineland Adaptive Behavior Scales (VABS) assesses adaptive behavior of individuals from birth to 90 years and encompasses five domains: communication (receptive, expressive, and written), daily living (personal care, domestic care, and community), socialization (interpersonal relationships, play and leisure time, and coping skills), physical activity/motor skills (gross and fine motor skills), and maladaptive behaviors (internalizing and externalizing behaviors) [19, 20]. Other measures of adaptive function used in MPS studies are the Behavior Assessment System for Children (BASC) [21] and the Scales of Independent Behavior, Revised (SIB-R) [22]. The BASC assesses the psychological adjustment and self-perceptions of children and provides clinical scales of emotional adjustment and adaptive behaviors (including parent rating scales, self-report of personality, and interview-based self-reports) [21]. The SIB-R measures 14 areas of adaptive behavior and 8 areas of problem behavior and can be used from infancy to over 80 years of age [23]. All of the above measures of adaptive function can be completed by either a parent or caregiver.

**HRQoL**

HRQoL is defined by the European Medicines Association as ‘the patient’s subjective perception of the impact of his disease and its treatment(s) on his daily life, physical, psychological and social functioning and wellbeing’[24, 25].

The best known generic HRQoL questionnaires are the General Health-Related Quality of EQ-5D questionnaire [9] and the Short-Form (SF)-36. These questionnaires are well validated, available in many languages and applicable to both patients with various diseases and healthy individuals. Both questionnaires have been used to assess HRQoL in MPS patients. The most recent version of the EQ-5D questionnaire (the EQ-5D-5L) is designed for self-completion in 3-5 minutes and comprises five dimensions (5D): mobility, self-care, usual activities, pain/discomfort and anxiety/depression. Each dimension has five levels (5L) coded from 1 to 5 (no to extreme problems). EQ-5D-5L health states can be converted into a single summary index value (utility), with a utility value of “1” representing perfect health and a value of “0” representing death [9]. Normalisation of the scale to a healthy population means that some MPS patients would provide ratings with negative values to suggest they feel worse than death. There are different versions of the EQ-5D for children (≥8 years) and for adults (≥16 years). A limitation of the EQ-5D is that it does not discriminate between anxiety and depression and does not collect data on psychological factors like anger, isolation, low self-esteem, self-harm, and suicidal ideation. A version of the EQ-5D in pictorial form would be valuable for persons with learning disability or cognitive impairment. The SF-36 has been developed for use in adults and older adolescents (≥16 years) and takes 10 minutes to complete [26]. It assesses eight subitems in two subdomains, i.e. the Physical Component Summary (PCS) and the Mental Component Summary (MCS). Each of the eight scales, as well as the two summary scores, are scored from 0 to 100, with higher scores indicating better QoL and a score of 50 (± 10 SD) being representative of the general population (i.e. norm-based scoring) [26, 27]. There is not information on how the EQ-5D and the SF-36 perform in patients with MPS.

The Health Utilities Index (HUI) is another generic instrument that has been used to assess the experience of MPS patients aged 5 years or older undergoing therapy, the long-term outcomes associated with disease or therapy, the efficacy, effectiveness and efficiency of health care interventions and health status [16].

Several MPS studies have used generic instruments to measure HRQoL in children, including the Pediatric Quality of Life inventory (PedsQL) [3, 28, 29], the Pediatric Evaluation of Disability Inventory (PEDI)[30, 31], the TNO-AZL Preschool children and Children QoL (TAPQOL and TACQOL) questionnaires [32, 33], and the childhood Health Questionnaire (CHQ)[16]. These validated instruments are specifically designed for use in children and are not suitable for adult patients. The 23-item PedsQL, appropriate in children and adolescents (< 18 years), consists of four multidimensional domains: physical, emotional, social and school functioning. Items are reverse-scored and linearly transformed to a 0–100 scale with higher scores indicating better HRQoL [3, 28]. The PEDI measures capability and performance in children from 6 months to 7.5 years in three categories: self-care, mobility and social function. According to the score obtained in each domain, the performance is classified as below normal (<30), normal (30-70) and above normal (>70) [30, 31]. The TAPQOL is a 43-item questionnaire consisting of 12 scales covering four domains (physical, social, cognitive and emotional functioning) developed for parents of patients from 6 months to 5 years [32, 34]. The TACQOL (for parents of and patients from 6-15 years) is a 56-item questionnaire consisting of seven domains (body, motor, autonomous, social and cognitive functioning and negative and positive moods) [33]. The presence of a specific limitation is scored on a 3-point scale (never-occasionally-often). If limitations are present, the wellbeing of the child related to that complaint is measured on a 4-point scale (fine-not so good-quite bad-bad). Scale scores are calculated by adding up item scores within scales, and transforming crude scales scores linearly to a 0-100 scale, with higher scores indicating better QoL [32, 33]. The CHQ assesses physical and psychosocial health status in children aged 5–18 years. It has been validated in a wide range of conditions, including MPS II. The version for children includes ten multi-item scales (physical functioning, role/social–emotional, role/social–behavioural, role/social–physical, bodily pain, general behaviour, mental health, self-esteem, general health perceptions, and family activities) and two single-item scales (change in health and Family Cohesion). The parent or caregiver form includes the 14 health concepts and HRQoL impacts included in the children version and two additional multi-item scales (parental impact-emotional and parental impact–time. Individual scale scores can be analysed separately, or combined to derive an overall physical and psychosocial score, ranging from 0 to 100, with higher scores indicating better health [16].

**Impact on caregivers**

The PedsQL Family Impact Module has been used in MPS II studies to assess the impact of a child’s chronic health condition on the parent and family during the past month [19, 29]. It consists of 36 items measuring the parent’s physical, emotional, social, and cognitive functioning, communication, worry, and problems specific to the family’s daily activities and family relationships. The questionnaire uses a 5-point Likert scale to evaluate the frequency of the child’s self-reported experiences for each item, ranging from 0 (never) to 4 (almost always) [35].

A study of patients with MPS IVA used the Zarit Burden Interview (ZBI), a validated questionnaire developed for caregivers of older patients [36]. The ZBI contains 22 items investigating the burden of caring in 5 domains: burden in the relationship, emotional well-being, social and family life, finances, and loss of control over one’s life, with a higher score indicating greater caring distress [37].

**References**

1. The Brief Pain Inventory. MD Anderson Cancer Society. http://www.mdanderson.org/education-and-research/departments-programs-and-labs/departments-and-divisions/symptom-research/symptom-assessment-tools/brief-pain-inventory.html. Accessed 21 April, 2016.

2.Ali N,Cagle S. Psychological health in adults with morquio syndrome. JIMD Rep. 2015;20:87-93.

3.Brands MMG, Güngör D, van den Hout JMP, Karstens FPJ, Oussoren E, Plug I, et al. Pain: a prevalent feature in patients with mucopolysaccharidosis. Results of a cross-sectional national survey. J Inherit Metab Dis. 2015;38:323-331.

4.Hendriksz CJ, Lavery C, Coker M, Ucar SK, Jain M, Bell L, et al. Burden of disease in patients with Morquio A syndrome: results from an international patient-reported outcomes survey. Orphanet J Rare Dis. 2014;9:32.

5. Breau LM, McGrath PJ, Camfield CS, Finley GA. Psychometric properties of the non-communicating children's pain checklist-revised. Pain 2002;99:349-57.

6.Swiedler SJ, Beck M, Bajbouj M, Giugliani R, Schwartz I, Harmatz P, et al. Threshold effect of urinary glycosaminoglycans and the walk test as indicators of disease progression in a survey of subjects with mucopolysaccharidosis VI (Maroteaux-Lamy syndrome). Am J Med Genet. 2005;134A:144-150.

7.Harmatz P, Ketteridge D, Giugliani R, Guffon N, Teles EL, Miranda MC, et al. Direct comparison of measures of endurance, mobility, and joint function during enzyme-replacement therapy of mucopolysaccharidosis VI (Maroteaux-Lamy syndrome): results after 48 weeks in a phase 2 open-label clinical study of recombinant human N-acetylgalactosamine 4-sulfatase. Pediatrics. 2005;115:e681-e689.

8. Jacob E, Mack AK, Savedra M, Van Cleve L, Wilkie DJ. Adolescent pediatric pain tool for multidimensional measurement of pain in children and adolescents. Pain Manag Nurs 2014;15:694-706.

9. EuroQol Group. EQ-5D-5L User Guide. http://www.euroqol.org/about-eq-5d/publications/user-guide.html. Accessed 21 April 2016 & http://www.euroqol.org/about-eq-5d/valuation-of-eq-5d.html. Accessed 21 April 2016

10. Shields BJ, Palermo TM, Powers JD, Grewe SD, Smith GA. Predictors of a child's ability to use a visual analogue scale. Child Care Health Dev 2003;29:281-90

11.Kuratsubo I, Suzuki Y, Orii KO, Kato T, Orii T,Kondo N. Psychological status of patients with mucopolysaccharidosis type II and their parents. Pediatr Int. 2009;51:41-47.

12. Ramey DR, Raynauld JP, Fries JF. The health assessment questionnaire 1992: status and review. Arthritis Care Res 1992;5:9-29.

13. Singh G, Athreya BH, Fries JF, Goldsmith DP. Measurement of health status in children with juvenile rheumatoid arthritis. Arthritis Rheum 1994;37:761-9.

14.Lysosomal Storage Disease Registry. https://www.lsdregistry.net/mpsiregistry/hcp/partic/mreg_hc_p_healthassess.asp. Accessed March 2014.

15.Hendriksz CJ, Giugliani R, Harmatz P, Mengel E, Guffon N, Valayannopoulos V, et al. Multi-domain impact of elosufase alfa in Morquio A syndrome in the pivotal phase III trial. Mol Genet Metab. 2015;114:178-185.

16.Raluy-Callado M, Chen WH, Whiteman DAH, Fang J,Wiklund I. The impact of Hunter syndrome (mucopolysaccharidosis type II) on health-related quality of life. Orphanet J Rare Dis. 2013;8:101.

17. Kato T, Kato Z, Kuratsubo I, Ota T, Orii T, Kondo N, Suzuki Y. Evaluation of ADL in patients with Hunter disease using FIM score. Brain Dev 2007;29:298-305.

18. Functional Independence Measurement (FIM) User Manual Version 1.0. 2003. http://www.va.gov/vdl/documents/clinical/func_indep_meas/fim_user_manual.pdf (last accessed June 2015).

19.Needham M, Packman W, Rappoport M, Quinn N, Cordova M, Macias S, et al. MPS II: adaptive behavior of patients and impact on the family system. J Genet Couns. 2014;23:330-338.

20.Kunin-Batson AS, Shapiro EG, Rudser KD, Lavery CA, Bjoraker KJ, Jones SA, et al. Long-term cognitive and functional outcomes in children with mucopolysaccharidosis (MPS)-IH (hurler syndrome) treated with hematopoietic cell transplantation. JIMD Rep. 2016;doi:10.1007/8904_2015_521.

21.Shapiro EG, Rudser K, Ahmed A, Steiner RD, Delaney KA, Yund B, et al. A longitudinal study of emotional adjustment, quality of life and adaptive function in attenuated MPS II. Mol Genet Metab Rep. 2016;7:32-39.

22.Holt JB, Poe MD,Escolar ML. Natural progression of neurological disease in mucopolysaccharidosis type II. Pediatrics. 2011;127:e1258-1265.

23. Scales of Independent Behavior-Revised. http://www.hmhco.com/hmh-assessments/other-clinical-assessments/sib-r#sthash.hZuzuTRo.dpuf. Accessed May 2016.

24. Committee for Medicinal Products for Human Use. Reflection paper on the regulatory guidance for the use of health-related quality of life (HRQL) measures in the evaluation of medicinal products (EMEA/CHMP/EWP/139391/2004). London, UK: European Medicines Agency, 2005.

25.Riazi A. Patient-reported outcome measures in multiple sclerosis. Int MS J. 2006;13:92-99.

26. SF-63.org. http://www.sf-36.org/tools/sf36.shtml. Accessed 21 April 2016.

27.Miller D, Rudick RA,Hutchinson M. Patient-centered outcomes: translating clinical efficacy into benefits on health-related quality of life. Neurology. 2010;74(Suppl 3):S24-S35.

28. Varni JW, Burwinkle TM. The PedsQL as a patient-reported outcome in children and adolescents with Attention-Deficit/Hyperactivity Disorder: a population-based study. Health Qual Life Outcomes 2006;4:26.

29.Needham M, Packman W, Quinn N, Rappoport M, Aoki C, Bostrom A, et al. Health-related quality of life in patients with MPS II. J Genet Couns. 2015;24:635-644.

30.Guarany NR, Schwartz IVD, Guarany FC,Giugliani R. Functional capacity evaluation of patients with mucopolysaccharidosis. J Pediatr Rehabil Med. 2012;5:37-46.

31.Beck M, Muenzer J,Scarpa M. Evaluation of disease severity in mucopolysaccharidoses. J Pediatr Rehabil Med. 2010;3:39-46.

32. Bunge EM, Essink-Bot ML, Kobussen MP, van Suijlekom-Smit LW, Moll HA, Raat H. Reliability and validity of health status measurement by the TAPQOL. Arch Dis Child 2005;90:351-8.

33. Verrips EGH, Vogels TGC, Koopman HM, Theunissen NCM, Kamphuis RP, Fekkes M, Wit JM, Verloove-Vanhorick SP. Measuring health-related quality of life in a child population. Eur J Public Health. 1999;9:188-193.

34.Brands MMMG, Oussoren E, Ruijter GJG, Vollebregt AAM, van den Hout HMP, Joosten KFM, et al. Up to five years experience with 11 mucopolysaccharidosis type VI patients. Mol Genet Metab. 2013;109:70-76.

35.Varni JW, Sherman SA, Burwinkle TM, Dickinson PE,Dixon P. The PedsQL™ family impact module: preliminary reliability and validity. Health Qual Life Outcomes. 2004;2:55.

36.Hendriksz CJ, Lavery C, Coker M, Kalkan Ucar S, Jain M, Bell L, et al. The burden endured by caregivers of patients with morquio a syndrome: results from an international patient-reported outcomes survey. JIEMS. 2014;doi:10.1177/2326409814540872.

37. Zarit SH, Reever KE, Back-Peterson J. Relatives of the impaired elderly: correlates of feelings of burden. Gerontologist. 1980;20:649-55.
